# Association of Age-Related Cataract With Skin Cancer in an Australian Population

**DOI:** 10.1167/iovs.61.5.48

**Published:** 2020-05-27

**Authors:** Shiwani Sharma, Catherine Lang, Jyoti Khadka, Maria C. Inacio

**Affiliations:** ^1^ Discipline of Ophthalmology, College of Medicine and Public Health, Flinders University, South Australia, Australia; ^2^ Registry of Senior Australians (ROSA), South Australian Health and Medical Research Institute (SAHMRI), Adelaide, South Australia, Australia; ^3^ Health and Social Care Economics Group, College of Nursing and Health Sciences, Flinders University, South Australia, Australia; ^4^ Institute for Choice, Business School, University of South Australia, South Australia, Australia; ^5^ Division of Health Sciences, University of South Australia, Adelaide, South Australia, Australia

**Keywords:** cataract, skin cancer, coexistence, population-based study, database analysis

## Abstract

**Purpose:**

Ultraviolet radiation from sunlight contributes to age-related cataract and skin cancer. The *EPHA2* gene is implicated in both these diseases. The purpose of this study was to determine whether age-related cataract and skin cancer are associated in a cohort of older Australians.

**Methods:**

A cross-sectional study was performed using the Historical Cohort of the Registry of Senior Australians. Individuals aged ≥65 years or aged ≥50 years and of Aboriginal or Torres Strait Islander descent, who had an aged care eligibility assessment between July 2005 and June 2015, and had a history of cataract surgery and/or skin cancer according to the Australian Government Medicare Benefits Schedule dataset, during the 3-year period prior, were evaluated (*N* = 599,316). A multivariable logistic regression model was used to determine association and multiple hypothesis correction was employed.

**Results:**

Of the evaluated individuals, 87,097 (14.5%) had a history of cataract and 170,251 (28.4%) a history of skin cancer. Among those with a history of cataract, 20,497 (23.5%), 1127 (1.3%), and 14,730 (16.9%) individuals had a concurrent history of keratinocyte, melanoma, and premalignant/solar keratosis, respectively. Those with a history of cataract were 19% more likely to have a history of skin cancer (odds ratio [OR], 1.19; 95% confidence interval [CI], (1.17–1.21). Co-occurrence of keratinocyte skin cancer was 16% (OR, 1.16; 95% CI, 1.14–1.18), melanoma 21% (OR, 1.21; 95% CI, 1.13–1.29), and premalignant/solar keratosis 19% (OR, 1.19; 95% CI, 1.17–1.22) more in the presence than absence of history of cataract.

**Conclusions:**

Age-related cataract is positively associated with skin cancer and its subtypes, including premalignant lesions in an older Australian population.

Cataract, a vision-impairing opacification of the ocular lens, is the leading cause of blindness worldwide.^1^^,^^2^ Age-related cataract, the most common form, occurs after the age of 50 years.^3^ Pediatric cataracts can occur in babies, infants, and children and are rare. The prevalence of age-related cataract increases with age from around 4% in those aged 55 to 64 years to >90% in those aged ≥80 years.^4^ In Australia, approximately 30% of individuals aged ≥50 years have age-related cataract, and the prevalence increases to approximately 80% in those aged ≥80 years.^5^ The prevalence is higher in Indigenous, with a rate that is 5 to 6 times higher in those aged ≤60 years, than non-Indigenous Australians.^6^

Age-related cataract is classified into nuclear, cortical, and posterior subcapsular cataract depending on the location of lens opacity. Nuclear or cortical cataract is the most prevalent form in most populations. Age-related cataract is a complex and multifactorial disease; both genetic and environmental factors contribute to the disease risk. Genetic variants, mainly single nucleotide polymorphisms, in the *EPHA2, WRN, KCNAB1*, and *CRYAA* genes, have been associated with increased risk of the disease.^7^^–^^10^ Genetic variation in the *EPHA2* gene that encodes a tyrosine kinase membrane receptor is the most reproducibly associated genetic risk factor for age-related cataract to date. Genetic variants in this gene are associated with the disease risk in multiple and ethnically diverse populations in the world, and with the risk of all forms of the disease.^7^^,^^8^^,^^11^ Environmental risk factors associated with the disease include older age, female gender, diabetes, hypertension, corticosteroid use, smoking, alcohol consumption, and exposure to ultraviolet radiation (UVR) such as from sunlight.^12^ Exposure to UVR from sunlight has been mainly associated with the risk of age-related cortical cataract.^13^^–^^16^ Climatic UVR has been positively correlated with cataract prevalence in the Indigenous Australian population.^6^

Skin cancer is the most common form of malignancy worldwide.^17^ Depending on the skin cell types involved, it is classified into malignant melanoma and nonmelanoma or keratinocyte skin cancer (KSC).^17^ Basal cell carcinoma (BCC) and squamous cell carcinoma (SCC) are the two major subtypes of KSC. KSC is more common and accounts for 96% of skin cancers; however, malignant melanoma is more aggressive and accounts for 65% of skin cancer-related deaths in the United States.^17^ The incidence of skin cancer is higher in white populations and in people with paler skin color, and higher in males than females.^17^ The incidence of KSC in the aging population reportedly increases with age, whereas malignant melanoma occurs in both young and older adults.^18^ The incidence of both malignant melanoma and KSC has been increasing worldwide.^19^ Australia has the highest incidence of KSC, particularly BCC, and the second highest incidence of melanoma skin cancer in the world.^19^ In Australia, the estimated prevalence of KSC was 2% in 2002, and the incidence has been reportedly increasing, with 3.3% of Medicare services provided for its treatment in 2011; the incidence of BCC is higher than that of SCC.^20^ In 2011, the estimated incidence of BCC was 2448 per 100,000 person-years.^19^ Similar increasing trends of melanoma skin cancer have been reported in Australia; in 2015, the age-standardized incidence rate was 52 cases per 100,000 persons.^21^ The incidence varies considerably across states and territories; in 2005–2009 it was the highest in Queensland and lowest in the Northern Territory with age-standardized incidence rate of 67 cases and 32 cases per 100,000, respectively.^22^ In 2014, melanoma skin cancer was one of the most common causes of cancer-related deaths in Australia.^23^ The majority of the Australian population (>85%) being of European descent is white and has higher risk of developing skin cancer.^24^^,^^25^

Exposure to UVR, mainly from sunlight, is the major cause of all types of skin cancer. Other risk factors include radiotherapy, indoor tanning, chemical exposure (e.g., to arsenic), long-term immunosuppression, and genetic factors. UVR-induced DNA damage and generation of reactive oxygen species leading to oxidative stress, macromolecule damage, and immunosuppression, are thought to underlie the pathogenesis.^17^ The *EPHA2* gene, a risk factor for age-related cataract, is overexpressed in several types of cancers including skin cancer, mainly malignant melanoma.^26^ Its downregulation and neutralization of the encoded protein have been associated with decreased migration and invasion of melanoma cells indicating its role in oncogenesis.^27^^,^^28^ *Epha2* knockout mice exhibit a greater susceptibility to chemically induced skin cancer, which suggests a tumor suppressor function of this gene, and its overexpression in tumor cells a likely compensatory mechanism to decrease cell proliferation.^29^ Regardless of the mechanism, *EPHA2* is involved in skin cancer.

UVR from sunlight and *EPHA2* gene play important roles, both in age-related cataract and skin cancer. Our work in mice showed that *Epha2* influences progression of UVR-induced cataract^30^ and UVR treatment has been reported to regulate *EPHA2* expression in skin cells,^31^ suggesting that UVR interacts with EPHA2 signaling in both cataract and skin cancer. Therefore, we hypothesized that these 2 diseases are associated and coexist. To test this hypothesis, in this study, we examined the association between history of age-related cataract and skin cancer in a large national cohort of older individuals accessing aged care services in Australia.

## Methods

Ethics approval for this study was received from the human research ethics committees of the University of South Australia, Adelaide (ID: 200489), and the Australian Institute of Health and Welfare (EO2018/1/418), before commencement of the study.

### Study Design, Setting, and Data Sources

A cross-sectional observational study was conducted using data from the National Historical Cohort of the Registry of Senior Australians. The Historical Registry of Senior Australians is a de-identified cohort of people who were assessed for or received aged care services in Australia between 1997 and 2017. This dataset contains information from the Australian Institute of Health and Welfare National Aged Care Data Clearinghouse datasets (including the National Death Index), the Australian Government Medicare Benefits Schedule (MBS), and the Pharmaceutical Benefits Scheme databases. The National Aged Care Data Clearinghouse Aged Care Assessment Program dataset was used to identify eligibility assessments for aged care services, which consisted of diagnosed health conditions and demographic information for individuals included in this study. The MBS dataset contains records of all health services that are subsidized by Medicare and was used to identify services provided for treatment of cataract and skin cancer to individuals included in this study. Finally, the Pharmaceutical Benefits Scheme dataset contains records of all prescription medications and was used to identify the comorbidity profile of the study participants.

### Study Sample

People aged ≥65 years or aged ≥50 years and of Aboriginal or Torres Strait Islander descent who had a first-time aged care eligibility assessment between July 1, 2005, and June 30, 2015, were included (*N* = 714,579). Individuals with Department of Veteran's Affairs cards (*n* = 108,687 [15%]) and those who did not have either of MBS or Pharmaceutical Benefits Scheme data (*n* = 6,576 [0.9%]) were excluded from the sample. The final sample included 599,316 individuals.

### Independent Variable

The independent variable in the analysis was history of cataract. This was determined using either of health condition “Cataract” (Aged Care Assessment Program code 702) reported in aged care eligibility assessment or a history of cataract surgery during a 3-year period prior, using the individual's MBS encounters with relevant procedures codes (Supplementary Table S1); a history of cataract surgery was limited to 3-year period before aged care eligibility assessment to include the maximum number of participants in the study.

### Dependent Variable

The dependent variable in the analysis was history of skin cancer. This variable was determined from either occurrence of ≥1 skin cancer removal procedures using MBS encounters (Supplementary Table S1) within a 3-year period before the participants' aged care eligibility assessment or from the reported health condition of “Skin cancer” (Aged Care Assessment Program code 205) in the aged care eligibility assessment itself. Skin cancer subtypes, including keratinocyte, melanoma, and premalignant/solar keratosis, were also examined and were determined from MBS procedures codes (Supplementary Table S1).^32^

### Covariates

Sex, age, country of birth (Australia vs. overseas), state of residence, Indigenous status (Aboriginal or Torres Strait Islander vs. non-Indigenous), non-skin cancers (yes/no), diabetes (yes/no), glaucoma (yes/no), and number of comorbidities were evaluated as confounders. Comorbidities were ascertained from the aged care eligibility assessment, the medication based comorbidity measure RxRisk-V,^33^ or a combination of both, for glaucoma, dementia, and diabetes.

### Statistical Analysis

Means, standard deviations, medians, interquartile ranges, frequencies, and proportions are used to describe the study sample. Logistic regression models were used to estimate the association of history of cataract surgery with that of skin cancer. Odds ratios (OR) and 95% confidence intervals (CI) are presented. Model fit was assessed using goodness of fit test. Models were adjusted for all the covariates. Colinearity between the variables in the final models was evaluated and tolerance values were >0.1 in all cases. All reported *P* values for main effects were considered statistically significant when <0.0125 (α = 0.05/4) using the conservative Bonferroni adjustment for multiple hypothesis testing conducted for the four dependent variables evaluated.^34^ In the adjusted models, 13,963 persons (2.3%) were excluded owing to missing data for covariates: Aboriginal or Torres Strait Islander status 11,144 (1.9%), country of birth 3057 (0.5%), and other variables <0.1%. SAS 9.4 (SAS Institute, Cary, NC) was used for all analysis.

## Results

Of the 599,316 individuals evaluated, 87,097 (14.5%) had a history of cataract and 170,251 (28.4%) had a history of skin cancer. The characteristics of the study sample by cataract status are shown in Table 1. Presence of history of cataract increased with age and 70.4% individuals with a positive history were ≥80 years old, although this age group constituted only 62.9% of the study cohort. Of those with a positive history of cataract, 66.2% were females. These trends are consistent with those reported for age-related cataract in large population studies.^4^ Of the individuals with a history of cataract, 28,075 (32.2%) also had a history of skin cancer (Table 2).

**Table 1.** Study Sample Characteristics by History of Cataract

	History of Cataract	
Characteristics	Yes, No. (%)	No, No. (%)
Total (from *n* = 599,316)	*n* = 87,097 (14.5)	*n* = 512,219 (85.5)
Age, y		
Median (IQR)	83 (79–87)	82 (76–87)
Under 70	2623 (3.0)	37,741 (7.4)
70–80	23,171 (26.6)	158,780 (31.0)
80–90	50,485 (58.0)	248,245 (48.5)
90 Plus	10,818 (12.4)	67,453 (13.2)
Female	57,655 (66.2)	321,407 (62.7)
Aboriginal/Torres Strait Islander	746 (0.9)	6464 (1.3)
Born in Australia	59,097 (67.9)	335,220 (65.4)
State		
NSW	31,360 (36.0)	178,296 (34.8)
VIC	22,133 (25.4)	126,113 (24.6)
QLD	16,588 (19.0)	96,460 (18.8)
SA	6726 (7.7)	47,385 (9.3)
WA	6564 (7.5)	42,841 (8.4)
TAS	2510 (2.9)	13,734 (2.7)
ACT	831 (1.0)	5177 (1.0)
NT	385 (0.4)	2213 (0.4)
No. of comorbidities		
0	3451 (4.0)	23,415 (4.6)
1–4	39,670 (45.5)	236,172 (46.1)
5–9	40,399 (46.4)	231,918 (45.3)
≥10	3577 (4.1)	20,714 (4.0)
Non-skin cancers	13,918 (16.0)	84,112 (16.4)
Diabetes	19,010 (21.8)	112,726 (22.0)
Glaucoma	13,891 (15.9)	55,510 (10.8)

ACT, Australian Capital Territory; IQR, interquartile range; NSW, New South Wales;

NT, Northern Territory; QLD, Queensland; SA, South Australia; TAS, Tasmania; VIC, Victoria; WA, Western Australia; y, years.

**Table 2.** Study Sample by History of Skin Cancer, Skin Cancer Subtypes, and History of Cataract

		**History of Cataract** ^*^	
	**Total Sample**	**Yes, No. (%)**	**No, No. (%)**
Total	599,316	87,097 (14.5)	512,219 (85.5)
Any history of skin cancer	170,251 (28.4)	28,075 (32.2)	142,176 (27.8)
Keratinocyte, primary or recurrent	124,240 (20.7)	20,497 (23.5)	103,743 (20.3)
Melanoma	6517 (1.1)	1127 (1.3)	5390 (1.1)
Premalignant/solar keratosis	87,423 (14.6)	14,730 (16.9)	72,693 (14.2)

* There is overlap between the groups owing to participants having multiple skin cancer treatment procedures. There were 5361 individuals who were diagnosed with skin cancer as listed in the aged care eligibility assessment but did not have a procedure for treatment of skin cancer; these individuals were not classified by skin cancer subtype.

Of those evaluated, there were 124,240 (20.7%) individuals with a history of keratinocyte, 6517 (1.1%) with a history of melanoma and 87,423 (14.6%) with a history of premalignant/solar keratosis (Table 2). Among those with a history of cataract, a concurrent history of keratinocyte was identified in 20,497 (23.5%), of melanoma in 1127 (1.3%), and of premalignant/solar keratosis in 14,730 (16.9%) individuals. A history of skin cancer was more prevalent in those with a history of cataract than those without (32.2% vs. 27.8%). The prevalence of history of primary or recurrent keratinocyte, melanoma, and premalignant/solar keratosis was also relatively higher in the presence than absence of history of cataract (23.5% vs. 20.3%, 1.3% vs. 1.1% and 16.9% vs. 14.2%, respectively).

After adjustment for covariates, there was a 1.19 (95% CI, 1.17–1.21) higher odds or chance of someone with a history of cataract to also have a history of skin cancer in this cohort (Table 3). The adjusted odds of someone with a history of cataract to also have a history of KSC was 1.16 (95% CI, 1.14–1.18) higher, to also have a history of melanoma was 1.21 (95% CI, 1.13–1.29) higher, and to also have a history of premalignant/solar keratosis was 1.19 (95% CI, 1.17–1.22) higher (Table 3).

**Table 3.** Association of History of Skin Cancer and Skin Cancer Subtypes With History of Cataract

Outcome Variable	Crude OR (95%CI)	Adjusted OR (95%CI)	*P* Value
Any history of skin cancer^*^	1.24 (1.22–1.26)	1.19 (1.17–1.21)	<0.001
Keratinocyte, primary or recurrent^†^	1.21 (1.19–1.23)	1.16 (1.14–1.28)	<0.001
Melanoma^‡^	1.23 (1.16–1.32)	1.21 (1.13–1.29)	<0.001
Premalignant/solar keratoses^§^	1.23 (1.21–1.26)	1.19 (1.17–1.22)	<0.001

* Model 1: *n* = 585,353, 2.3% missing data. Model adjusted for sex, age, country of birth, state of residence, Indigenous status, nonskin cancers, diabetes, glaucoma, and comorbidity burden.

† Model 2: *n* = 585,353, 2.3% missing data. Model adjustment for covariates same as 1.

‡ Model 3: *n* = 585,353, 2.3% missing data. Model adjustment for covariates same as 1.

§ Model 4: *n* = 545,353, 2.5% missing data. Model adjustment for covariates same as 1.

Of the evaluated individuals, 377,001 were >80 years of age and 222,315 were <80 years of age. The prevalence of history of cataract and history of skin cancer including skin cancer subtypes and presence of concurrent history of both the diseases was higher among those >80 years old than those <80 years old (Supplementary Table S2). However, the adjusted odds of association of history of cataract with history of skin cancer and with history of each of the skin cancer subtypes were similar in those >80 and <80 years old (Supplementary Table S3).

## Discussion

Exposure to UVR mainly from sunlight is a well-recognized risk factor for age-related cataract and skin cancer. In this study, we found a positive association between these two diseases. This finding supports our hypothesis and suggests that these diseases share common underlying causes, such as UVR exposure. Relatively higher ambient UVR levels from sunlight are experienced in Australia, which supports UVR exposure as one of the underlying causes and positive association between the two diseases in the Australian population. According to this study, cataract is positively associated with both KSC and malignant melanoma, as well as with premalignant lesions or solar keratosis. Different patterns of UVR exposure are thought to cause BCC and SCC that constitute KSC, and to malignant melanoma; nevertheless, UVR is involved.^17^^,^^19^^,^^35^ A positive association of cataract with the main skin cancer subtypes also supports UVR to be a common underlying cause between the two diseases.

To date, only one other study, performed in an Israeli population, investigated association of cataract with skin cancer. The findings of this and the reported study are similar. Varssano et al.,^36^ using a local healthcare services database in individuals aged ≥40 years, performed a cross-sectional study and reported positive association of BCC, SCC, and actinic keratosis, the premalignant skin lesion from sun exposure, with increased likelihood of prevalent cataract. An association between melanoma and prevalent cataract was not found in that study despite a larger sample size than the present study. This difference between the two studies can be attributed to differences in the study populations and ambient solar UVR levels.

UVR is mainly known to increase the risk of age-related cortical cataract.^14^ The present study cohort likely includes all subtypes of age-related cataract because, in older Australians, the incidence of cataract surgery is relatively higher for age-related posterior subcapsular and nuclear cataract than for cortical cataract.^37^^,^^38^ Thus, the association of any cataract with skin cancer suggests that in addition to UVR these diseases likely share other common underlying cause(s). Because both age-related cataract and skin cancer are multifactorial diseases, it is possible that they share multiple common underlying causes, genetic and/or environmental. Each factor may have a small effect or contribute to the risk in a small number of cases or disease subtypes. A positive association between these diseases found in the Australian population in this study and reported in the Israeli population^36^ opens up the opportunity for identification of those causes. An investigation of the association between cataract and skin cancer in other populations in other parts of the world would indicate whether disease subtype and population-specific common underlying causes are involved in these diseases.

This study has several limitations. Because it is an observational study that relies on the linkage of three data sources, it could suffer from missing data, incorrect linkage, and miscoding. We attempted to minimize these issues by conducting several logic checks, excluding the cases with no linkage records (<1%), and checking whether the missing data were differential between the groups compared. It is likely that the history of cataract and skin cancer is underascertained in this cohort. The MBS dataset used for determining procedures performed for treatment of cataract and skin cancer does not include services provided to public patients in public hospitals or private services fully covered by the Department of Veteran's Affairs program. We know that, in 2014–2015, nationally, 71% of cataract surgeries performed were for private patients captured by the MBS and 29% for public patients.^39^ For skin cancer, MBS data captures all but 1.6% of procedures and prevalence estimates from these data have been shown to differ from other sources for those aged ≥80 years.^32^ To address this limitation, individuals who received health care services under the Department of Veteran's Affairs program were excluded from the study.^40^ Additionally, the aged care eligibility assessment used to ascertain the history of skin cancer and cataract only records up to 10 health conditions; the aged care eligibility assessors only report those conditions that have been diagnosed by a suitably qualified individual and impact on the person's need for assistance.^41^ It is possible that neither cataract nor skin cancer were listed. Because only 3% of people report the maximum number of 10 conditions, it is unlikely that underascertainment was due to the limit on the number of health conditions recorded, and more likely that these were not acute conditions or did not impact the need for assistance. Therefore, we also used the MBS service provision to identify procedures performed to address these conditions. Furthermore, cortical cataract is the most common cataract subtype associated with UVR, but it is less likely to cause visual impairment and require cataract surgery.^42^ Hence, there may be a significant under-ascertainment of this type of cataract in our cohort because it would not be captured in the MBS dataset and recorded in the aged care eligibility assessment. Cataract was not distinguishable by age of onset into early-onset or age-related cataract. However, a history of cataract surgery during the 3-year period before the aged care eligibility assessment, one of the inclusion criteria, suggested that cataract in this population was mainly age related. Another limitation of this work is that we were unable to examine the studied effect in the Indigenous Australian population, which is known to have higher prevalence of cataract^6^^,^^43^ and, according to limited number of studies, a lower incidence of melanoma skin cancer^6^^,^^44^; anecdotal evidence suggests rare occurrence of KSC in this population.^45^ An independent study with appropriate ethical and respectful community engagement and a large sample size is required to determine prevalence of skin cancer and its association with cataract in this population.

Despite these limitations and the potential underascertainment of history of cataract and skin cancer in the study cohort, we discovered a positive association between these diseases. This finding suggests that actually an even greater association may exist between these diseases in the older Australian population. Further research is warranted to determine association of age-related cortical cataract with skin cancer in the older Australian population.

This large epidemiological study also has several strengths, including its sample size, population-based coverage, and comprehensive systematic data collection. Our cohort captures a nationally representative sample of individuals accessing aged care services for which an aged care eligibility assessment is required in Australia; in the 2015–2016 financial year, the number of individuals accessing these services was, approximately 5.6% of the population >65 years of age.^46^ Our study relied on the systematic data collection that occurs as part of the comprehensive eligibility assessments performed in Australia for all individuals accessing aged care services; these assessments are performed by trained assessors. Additionally, our cohort contained the age range that is most at risk for cataract and skin cancer.

In conclusion, this large study of an older Australian cohort revealed a positive association of prevalent cataract with skin cancer and its main subtypes. Further research is warranted to determine which subtypes of cataract are associated with skin cancer in the Australian population. Inclusion of codes of cataract subtypes in the MBS database would facilitate subtype-specific analysis in similar studies in the future. Nevertheless, this study suggests that occurrence of cataract can be an indicator of occurrence of skin cancer and vice versa. Additionally, collaboration between ophthalmology and dermatology specialists can be beneficial for management of these diseases.

## Supplementary Material

Supplement 1

Supplement 2

Supplement 3
