# Supplementary material for: Association of Age-Related Cataract With Skin Cancer in an Australian Population
Source: Invest Ophthalmol Vis Sci. 2020 May 27;61(5):48. doi: 10.1167/iovs.61.5.48 (PMC7405762; doi:10.1167/iovs.61.5.48)
Supplement: Supplement 1 [file iovs-61-5-48_s001.pdf]

Supplementary material

Table S1. Medicare Benefits Schedule Codes used for determination of Skin Cancer Subtypes and Cataract Procedures

Condition	MBS item numbers	Additional Description
**Cataract**	42698, 42701, 42702, 42718	
**Skin Cancer: Keratinocyte**	31255,31260,31265, 31270,31275,31280, 31285, 31290	Surgical initial excision of a primary basal cell carcinoma (BCC) or squamous cell carcinoma (SCC), pathology confirmed
	30196,30197,30202, 30203, 30205	Malignant neoplasm of skin by pathology or specialist opinion confirmed, and treatment with cryotherapy or serial curettage excision
	31000, 31001, 31002	Mohs micro surgery of skin tumour & histology exam, Histological proof of malignancy
	31256,31261,31266, 31271,31276,31281, 31286, 31291, 31257,31262,31267, 31272,31277,31282, 31287, 31292, 31258,31263,31268, 31273,31278,31283, 31288, 31293, 31295	Residual and recurrent BCC and SCC
**Skin Cancer: Melanoma**	31300,31305,31310, 31315,31320,31325, 31330, 31335	Malignant melanoma and locally aggressive skin tumours, definitive surgical excision with an adequate surgical removal at that site
**Skin Cancer: Pre-malignant solar keratosis**	30192	Pre-malignant lesions, solar keratosis by ablative technique i.e. cryotherapy
	31200	Removal of skin lesions (Pre-malignant lesions, including solar keratosis)

MBS=Medicare Benefits Schedule. BCC=basal cell carcinoma. SCC=squamous cell carcinoma. Note: Medicare codes for skin cancer were restructured in November 2016. The codes in this table are the original codes used at the time of the procedure.
