# Supplementary material for: Association of Age-Related Cataract With Skin Cancer in an Australian Population
Source: Invest Ophthalmol Vis Sci. 2020 May 27;61(5):48. doi: 10.1167/iovs.61.5.48 (PMC7405762; doi:10.1167/iovs.61.5.48)
Supplement: Supplement 2 [file iovs-61-5-48_s002.pdf]

Table S2. Age stratified study sample by history of skin cancer, skin cancer subtypes and history of cataract

AGE ABOVE 80 YEARS

		**History of cataract***	
	**Total Sample**	**Yes**<br>**N (%)**	**No**<br>**N (%)**
**Total**	377,001	61,303	315,698
Any history of skin cancer	117,761(31.2)	20,940(34.2)	96,821(30.7)
Keratinocyte, primary or recurrent	87,686(23.3)	15,486(25.3)	72,200(22.9)
Melanoma	4,360(1.2)	817(1.3)	3,543(1.1)
Premalignant/ solar keratosis	58,975(15.6)	10,725(17.5)	48,250(15.3)

AGE UNDER 80 YEARS

		**History of cataract***	
	**Total Sample**	**Yes**<br>**N (%)**	**No**<br>**N (%)**
**Total**	222,315	25,794	196,521
Any history of skin cancer	52,490(23.6)	7,135(27.7)	45,355(23.1)
Keratinocyte, primary or recurrent	36,554(16.4)	5,011(19.4)	31,543(16.1)
Melanoma	2,157(1.0)	310(1.2)	1,847(0.9)
Premalignant/ solar keratosis	28,448(12.8)	4,005(15.5)	24,443(12.4)

* There is overlap between the groups due to participants having multiple skin cancer treatment procedures. There were 3,779 individuals aged above 80 years and 1,582 under 80 years who were diagnosed with skin cancer as listed in the aged care eligibility assessment but did not have a procedure for treatment of skin cancer; these individuals were not classified by skin cancer subtype.
