# Supplementary material for: Association of Age-Related Cataract With Skin Cancer in an Australian Population
Source: Invest Ophthalmol Vis Sci. 2020 May 27;61(5):48. doi: 10.1167/iovs.61.5.48 (PMC7405762; doi:10.1167/iovs.61.5.48)
Supplement: Supplement 3 [file iovs-61-5-48_s003.pdf]

Table S3. Association of history of skin cancer and skin cancer subtypes with history of cataract stratified by age

AGE ABOVE 80 YEARS

Outcome variable	Crude OR (95%CI)	Adjusted OR (95%CI)	P value
Any history of skin cancer[a]	1.17(1.15,1.19)	1.17(1.15,1.20)	<0.001
Keratinocyte, primary or recurrent[b]	1.14(1.12,1.16)	1.14(1.12,1.17)	<0.001
Melanoma[c]	1.19(1.10,1.29)	1.17(1.09,1.27)	<0.001
Premalignant/solar keratoses[d]	1.18(1.15,1.20)	1.16(1.14,1.19)	<0.001

OR=Odds ratio. CI=Confidence intervals.
[a]Model 1: N=369,522, 2.0% missing data. Model adjusted for sex, age, country of birth, state of residence, Indigenous status, non-skin cancers, diabetes, glaucoma, and co-morbidity burden.
[b]Model 2: N=369,522, 2.0% missing data. Model adjustment for covariates same as 1.
[c]Model 3: N=369,522, 2.0% missing data. Model adjustment for covariates same as 1.
[d]Model 4: N=369,522, 2.0% missing data. Model adjustment for covariates same as 1.

AGE UNDER 80 YEARS

Outcome variable	Crude OR (95%CI)	Adjusted OR (95%CI)	P value
Any history of skin cancer[a]	1.28(1.24,1.31)	1.16(1.12,1.20)	<0.001
Keratinocyte, primary or recurrent[b]	1.26(1.22,1.30)	1.14(1.10,1.18)	<0.001
Melanoma[c]	1.28(1.14,1.45)	1.21(1.07,1.37)	0.003
Premalignant/solar keratoses[d]	1.29(1.25,1.34)	1.18(1.14,1.12)	<0.001

OR=Odds ratio. CI=Confidence intervals.
[a]Model 1: N=215,831, 2.9% missing data. Model adjusted for sex, age, country of birth, state of residence, Indigenous status, non-skin cancers, diabetes, glaucoma, and co-morbidity burden.
[b]Model 2: N=215,831, 2.9% missing data. Model adjustment for covariates same as 1.
[c]Model 3: N=215,831, 2.9% missing data. Model adjustment for covariates same as 1.
[d]Model 4: N=215,831, 2.9% missing data. Model adjustment for covariates same as 1.
